# Visualizing Antimicrobials in Bacterial Biofilms: Three-Dimensional Biochemical Imaging Using TOF-SIMS

**DOI:** 10.1128/mSphere.00211-17

**Published:** 2017-07-19

**Authors:** Sarah K. Davies, Sarah Fearn, Luke P. Allsopp, Freya Harrison, Ecaterina Ware, Stephen P. Diggle, Alain Filloux, David S. McPhail, Jacob G. Bundy

**Affiliations:** a Department of Surgery and Cancer, Imperial College London, London, United Kingdom; b Department of Materials, Imperial College London, London, United Kingdom; c Department of Life Sciences, MRC Centre for Molecular Bacteriology and Infection, London, United Kingdom; d Centre for Biomolecular Sciences, School of Life Sciences, University of Nottingham, Nottingham, United Kingdom; e School of Biological Sciences, Georgia Institute of Technology, Atlanta, Georgia, USA; Providence Portland Medical Center

**Keywords:** *Pseudomonas aeruginosa*, TOF-SIMS, biofilms, imaging

## Abstract

Bacterial biofilms are groups of bacteria that exist within a self-produced extracellular matrix, adhering to each other and usually to a surface. They grow on medical equipment and inserts such as catheters and are responsible for many persistent infections throughout the body, as they can have high resistance to many antimicrobials. *Pseudomonas aeruginosa* is an opportunistic pathogen that can cause both acute and chronic infections and is used as a model for research into biofilms. Direct biochemical methods of imaging of molecules in bacterial biofilms are of high value in gaining a better understanding of the fundamental biology of biofilms and biochemical gradients within them. Time of flight–secondary-ion mass spectrometry (TOF-SIMS) is one approach, which combines relatively high spatial resolution and sensitivity and can perform depth profiling analysis. It has been used to analyze bacterial biofilms but has not yet been used to study the distribution of antimicrobials (including antibiotics and the antimicrobial metal gallium) within biofilms. Here we compared two methods of imaging of the interior structure of *P. aeruginosa* in biological samples using TOF-SIMS, looking at both antimicrobials and endogenous biochemicals: cryosectioning of tissue samples and depth profiling to give pseudo-three-dimensional (pseudo-3D) images. The sample types included both simple biofilms grown on glass slides and bacteria growing in tissues in an *ex vivo* pig lung model. The two techniques for the 3D imaging of biofilms are potentially valuable complementary tools for analyzing bacterial infection.

**IMPORTANCE** Modern analytical techniques are becoming increasingly important in the life sciences; imaging mass spectrometry offers the opportunity to gain unprecedented amounts of information on the distribution of chemicals in samples—both xenobiotics and endogenous compounds. In particular, simultaneous imaging of antibiotics (and other antimicrobial compounds) and bacterium-derived metabolites in complex biological samples could be very important in the future for helping to understand how sample matrices impact the survival of bacteria under antibiotic challenge. We have shown that an imaging mass spectrometric technique, TOF-SIMS, will be potentially extremely valuable for this kind of research in the future.

## Abstract (precis)

Modern analytical techniques are becoming increasingly important in the life sciences; imaging mass spectrometry offers the opportunity to gain unprecedented amounts of information on the distribution of chemicals in samples—both xenobiotics and endogenous compounds. In particular, simultaneous imaging of antibiotics (and other antimicrobial compounds) and bacterium-derived metabolites in complex biological samples could be very important in the future for helping to understand how sample matrices impact the survival of bacteria under antibiotic challenge. We have shown that an imaging mass spectrometric technique, TOF-SIMS, will be potentially extremely valuable for this kind of research in the future.

## INTRODUCTION

Time of flight (TOF)–secondary-ion mass spectrometry (SIMS) has many potential applications and, in part because of recent advances in instrumentation such as gas cluster ion beam (GCIB) technology, is increasingly being used for biological sample analysis (1, 2).

SIMS offers two main advantages over other mass spectrometry imaging techniques more commonly used for biological samples, such as matrix-assisted laser desorption ionization (MALDI) and desorption electrospray ionization (DESI). The first is its high maximum spatial resolution—lateral resolution can be as good as 200 nm in an optimal sample; however, this is rarely achieved in biological samples—compared with ~5 and ~35 μm with MALDI and DESI, respectively (reviewed by Spengler [2]), allowing analyses at the multicellular and single-colony levels. The second is the potential to acquire sequential mass spectra by ablating layers of the sample with an incident ion beam, allowing the reconstruction of pseudo-three-dimensional (pseudo-3D) images. Examples of depth profiling of biological samples include the use of a C_60_ beam by Vaidyanathan et al. to sputter into a *Streptomyces coelicolor* population to evaluate the distribution of two endogenous antibiotic compounds (3); they found that one compound was found solely on the cell surface and not within the cells. Sputtering has also been used to look at *Xenopus* oocytes (4), rat kidney samples and single HeLa cells (5), and fibroblasts (6). Depth profiling with TOF-SIMS has recently been applied to *in situ* imaging of *Shewanella oneidensis* biofilms grown in microchannels (7–9).

Biofilms are complex 3D structures produced as microorganisms create their own habitats (10–12). Groups of cells produce and are surrounded by an extracellular polymeric substance (EPS) matrix, adhering to each other and often to a surface (13). TOF-SIMS has been used previously to analyze *Pseudomonas aeruginosa* biofilms (9, 14, 15). The main group of endogenous metabolites looked at in previous work is quorum-sensing signaling molecules, which play a role in biofilm formation and virulence, specifically quinolones. *P. aeruginosa* produces >50 alkylquinolones that have a role in both cell-to-cell signaling and antibiotic activity (16, 17). Quinolones have also been found to have a role in interspecies and interkingdom communication (18). Recent work has used depth profiling in an attempt to look at the 3D structure of biofilm (9), but SIMS is still not a routine tool for imaging the biofilm interior using 3D techniques.

Cells that exist as part of a biofilm are much less susceptible to antimicrobials than planktonic cells are. Biofilms commonly grow on medical equipment and inserts such as catheters and, because of their resistance to antimicrobials, are often extremely difficult to remove (19). Bacteria also grow as biofilms within host tissues during chronic infection (20). *P. aeruginosa* biofilms are of particular concern, as they grow in the lungs of cystic fibrosis patients, causing highly persistent, antibiotic-resistant infections that are the primary cause of death for these patients (21, 22). It has been shown that some antimicrobials are sequestered to the periphery and do not penetrate the biofilm (23). Hall-Stoodley et al. (19) have summarized three proposed mechanisms to explain why biofilms are resistant to antimicrobials. The first is based on the idea that while many antibiotics can freely penetrate the EPS, cells within the biofilm are often still protected. This is possibly due to the fact that these inner cells exist in starved, stationary-phase, dormant pockets and most antibiotics depend on cellular activity. The second mechanism involves the presence of “persisters”—resistant cells—within the biofilm, comprising only a small part of the biofilm as a whole. The third mechanism involves the barrier formed by the EPS matrix. The EPS may bind larger antimicrobials such as immunoglobulins or neutralize charged agents like metals so that they are essentially diluted and fewer cells within the biofilm are reached. Clearly, direct biochemical analyses of biofilms could be of huge value in evaluating these different hypotheses. Standard approaches in the life sciences include using fluorescent labeling together with confocal microscopy. Fluorescence microscopy is clearly vastly powerful, and using modified fusion proteins allows interrogation of biochemical environments in living cells in three dimensions. However, there are also some limitations to this; in particular, for imaging of small-molecule antibiotics and other antimicrobials, it may not be possible to add a fluorescent tag without changing the analyte’s activity and distribution.

We took two approaches to the imaging of biofilms in cross section. In our first model, we grew biofilms on a conductive flat surface well suited to SIMS analysis and used TOF-SIMS in dynamic, depth-profiling mode to reconstruct pseudo-3D images of the biofilm. In our second model, we used *ex vivo* pig lung tissue as a biofilm substrate that would be both biologically relevant (24) and suitable for sectioning. We grew bacteria in association with lung tissue samples and used cryosectioning and 2D imaging with TOF-SIMS to analyze them. In both models, we focused on specific analytes, including endogenous biomolecules (quinolone signaling molecules) and xenobiotics (antimicrobials, including antibiotic small molecules, and gallium, an antimicrobial metal ion that has been shown to be effective in preventing the formation of biofilms [25]). We demonstrate that TOF-SIMS can be used to image endogenous and exogenous biofilm compounds, giving biochemical information on biofilm interiors.

## RESULTS AND DISCUSSION

### Endogenous bacterium-specific molecules can be identified in complex organic matrices, including biofilms and infected tissue.

**FIG 1:**
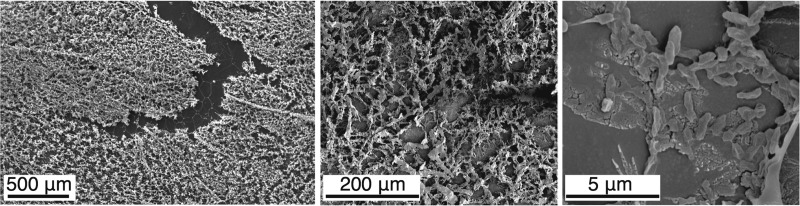
SEM images of biofilms grown on ITO-coated glass. Images are of a representative sample and are shown at magnifications of ×50, ×250, and ×10,000. Both cells and the extracellular matrix can be seen, confirming biofilm formation.

**FIG 2:**
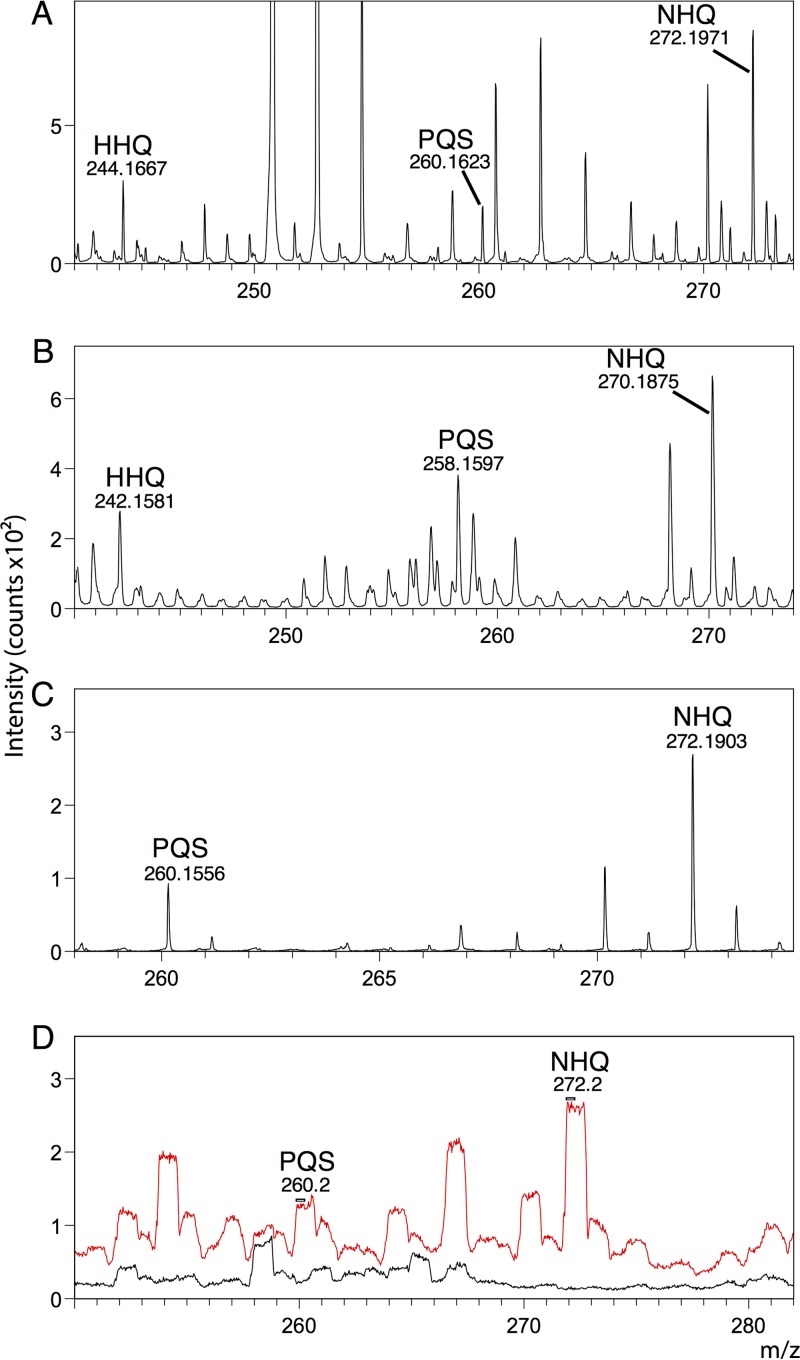
Putative assignment of (M + H)^+^ and (M − H)^−^ peaks in biofilms grown on ITO-coated glass for the quinolones HHQ (C_16_H_22_NO), PQS (C_16_H_21_NO_2_), and NHQ (C_18_H_25_NO) in positive-ion (A) and negative-ion (B) modes. We observed peaks for PQS and NHQ in infected pig bronchus samples in positive-ion mode (C) in HCBM and in low mass resolution imaging mode, BAM (D). PQS and NHQ were present in infected samples (red) but not in mock-infected control samples (black).

Bacteria grew as authentic biofilms on indium-tin-oxide (ITO)-coated glass slides with cells embedded in an extracellular matrix (Fig. 1). We identified multiple quinolones in both sample types on the basis of previously published values (14). We observed (M + H)^+^ and peaks corresponding to 2-heptyl-3-hydroxyquinolone (PQS) and 2-nonyl-4-quinolone (NHQ) in both sample types and additionally 2-heptyl-4-quinolone (HHQ) in ITO-coated glass samples (Fig. 2A, C, and D). We also putatively identified (M − H)^−^ peaks for HHQ, PQS, and NHQ in ITO-coated glass samples (Fig. 2B). Quinolone peaks were not present in mock infection controls in the bronchus model (Fig. 2D).

HHQ and PQS are generally accepted as the key quorum-sensing quinolones, but *P. aeruginosa* produces >50 alkylquinolones with multiple activities, including cell-cell signaling, antimicrobial effects, and virulence control (reviewed by Heeb et al. [17]). PQS is found in the lungs of cystic fibrosis patients with *P. aeruginosa* infections (26) and is able to inhibit the growth of other bacterial species common in cystic fibrosis patients (27).

### Quinolones are mostly distributed evenly throughout the biofilm but with some hot spots.

**FIG 3:**
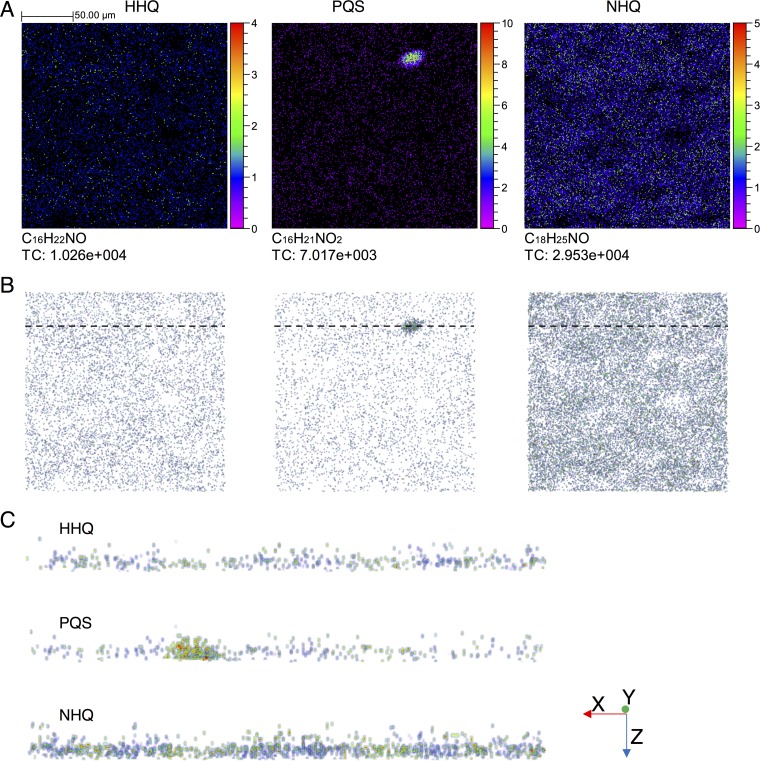
Distribution of HHQ, PQS, and NHQ and total counts (TC) in a 200- by 200-μm area of a *P. aeruginosa* biofilm grown on an ITO-coated glass substrate (A). Depth profiling was carried out so that 3D images could be reconstructed. The equivalent *x*-*y* images generated from the 3D reconstruction are shown in panel B, and virtual sections of 10 μm through the biofilm at an area with a high PQS concentration (indicated by a dashed line in panel B) are shown in panel C. Note that the *z* axis is shown on a relative scale (number of scans) rather than an absolute-distance scale.

We mapped the distribution of the three identifiable quinolones over a 200- by 200-μm area, sputtering through to the substrate surface to reconstruct a 3D biofilm image. NHQ—the most abundant quinolone—and HHQ appear to be homogeneously distributed across the area analyzed, while PQS is concentrated in one region (Fig. 3). This PQS hot spot is not an area with a higher HHQ or NHQ concentration, so this is unlikely to be simply an area of high cell density. We took a virtual section of 10 μm through the center of this area with a high PQS concentration and looked at the distribution of all three quinolones throughout the depth of the biofilm (Fig. 3C). This approach reveals some heterogeneity along the *x* axis for all quinolones, but hot spots—higher-concentration areas—are maintained as high-concentration areas through to the substrate surface, with no clear variation on the *z* axis.

HHQ, identified in the glass slide model, is the direct precursor of PQS (28). It may be that here the biofilms have not matured to the point at which PQS is widespread throughout the biofilm; cells release HHQ for adjacent cells to take up and convert to PQS. The differential distribution of quinolones on the biofilm surface and the presence of high-concentration hot spots are consistent with previous TOF-SIMS analyses of *P. aeruginosa* biofilms (14, 15); however, we have shown that these high-concentration areas are present through to the substrate surface.

### Quinolones can be imaged in infected mammalian tissue.

**FIG 4:**
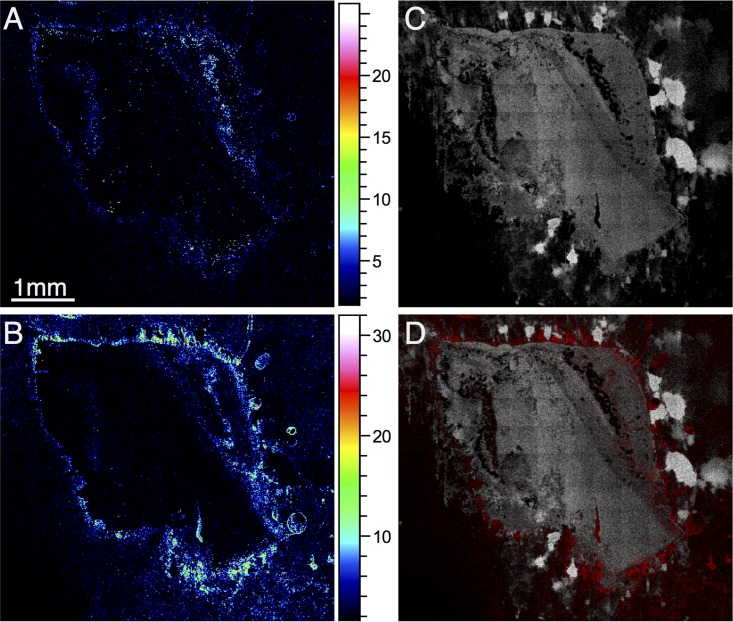
Distribution of signaling molecules in a section of *P. aeruginosa*-infected bronchus analyzed in positive-ion mode. PQS (total cell count, 1.239 × 10^5^) and NHQ (total cell count, 2.657 × 10^5^) are shown in panels A and B, respectively. An ion representative of bronchial tissue is shown in white in panel C (*m/z* 88.01; total cell count, 2.621 × 10^6^). The combined signal derived from PQS and NHQ is shown in red in panel D overlaid with *m/z* 88.01 in white.

We were able to section the frozen lung tissue samples without any embedding medium to avoid unwanted peaks from commonly used embedding substances (issues that may arise from certain sample preparation methods before TOF-SIMS analysis were discussed by Gamble et al. [29]). We identified both PQS and NHQ in the bronchial sections; these ions were present in infected tissue only and not in control samples (Fig. 2D). Of course, as these metabolites are being assigned purely on the basis of their *m/z* value, in combination with their appearing only in infected samples, these remain, to some degree, putative assignments. However, other TOF-SIMS studies have also assigned these compounds, and therefore, we believe that these are justifiable. Because of significant overlapping of HHQ peaks with peaks from bronchial tissue, we were unable to reliably identify HHQ in this model. Both PQS and NHQ penetrate the tissue to some degree but are mostly located around the perimeter of the bronchial section (Fig. 4). As in the glass substrate model, the total ion count was higher for NHQ than for PQS.

### Exogenous antimicrobials can be identified within the biofilm matrix.

**FIG 5:**
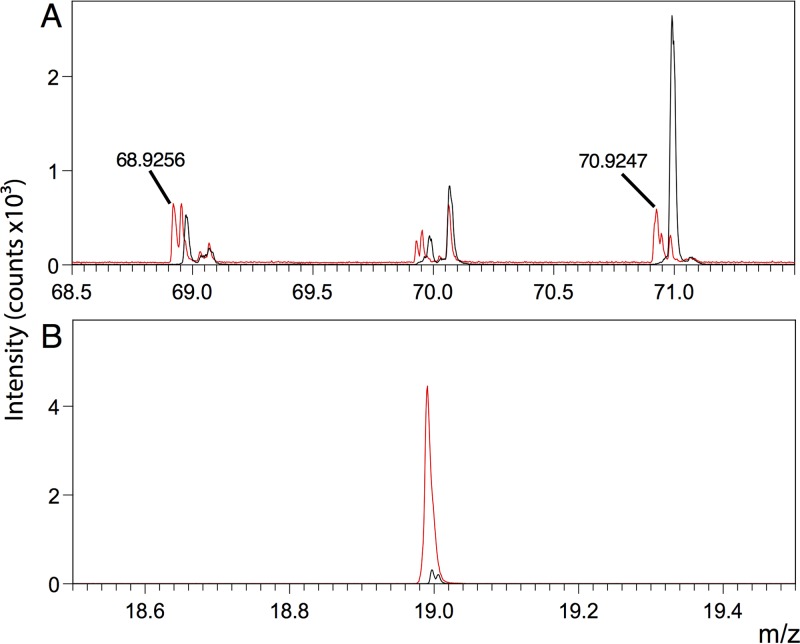
Gallium isotopes at *m/z* 68.9256 and 70.9247 are present in biofilms exposed to 5 μg/ml gallium nitrate in positive-ion mode (A). F^−^ is present in biofilms exposed to ciprofloxacin (C_17_H_18_FN_3_O_3_) at 20 μg/ml in negative-ion mode (B). Spectra from samples exposed to the antimicrobials are red; spectra from control samples (biofilms not exposed to antimicrobials) are black.

**FIG 6:**
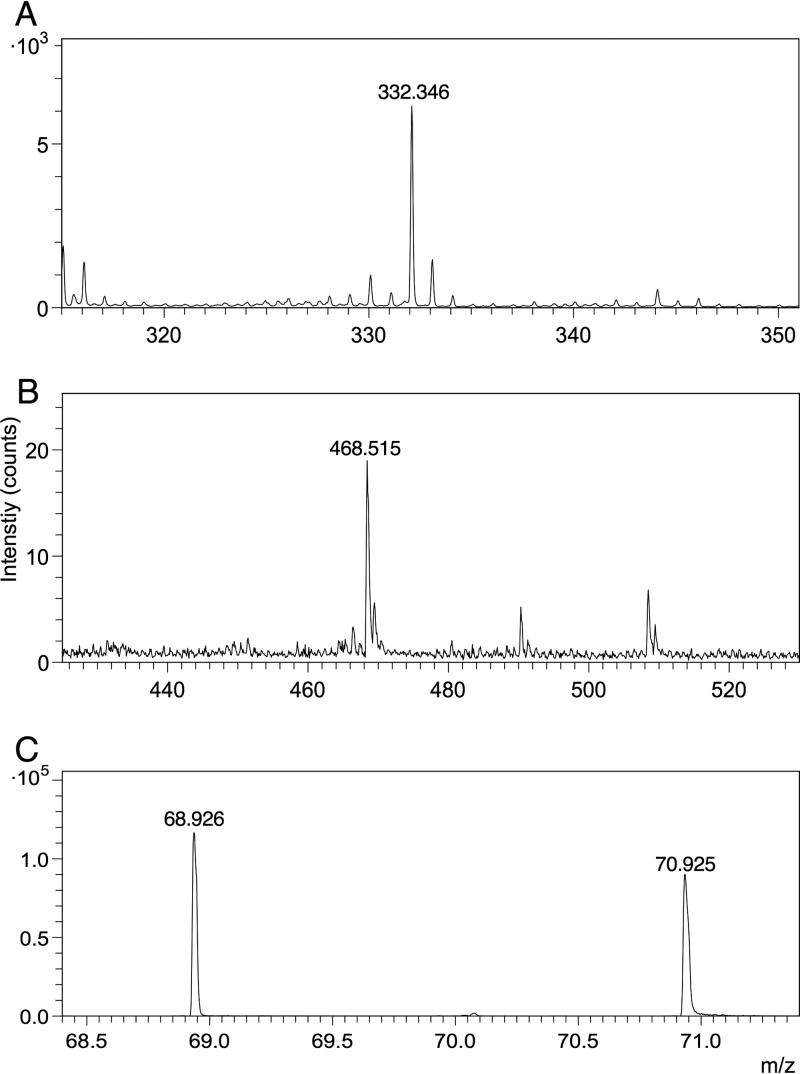
(M + H)^+^ parent ion peaks in ciprofloxacin (C_17_H_18_FN_3_O_3_) (A), tobramycin (C_18_H_37_N_5_O_9_) (B), and gallium (C) standards.

We exposed biofilms grown on ITO-coated glass to three clinically relevant antimicrobials (gallium nitrate, ciprofloxacin, and tobramycin) and were able to see these exogenous compounds within the biological matrix. We could see both gallium isotopes in standard spectra and within biofilm samples in positive-ion mode (Fig. 5A). We observed parent ion peaks and fragments for both ciprofloxacin (C_17_H_18_FN_3_O_3_, *m/z* 332.346) and tobramycin (C_18_H_37_N_5_O_9_, *m/z* 468.515) in standard spectra in positive-ion mode (Fig. 6). However, we were unable to reliably detect the tobramycin parent ion or fragments in biological samples because of a large number of biofilm peaks within the spectral regions of interest. Similarly, we could not reliably detect the parent ion or the majority of fragments from ciprofloxacin within the biofilm; however, we could detect F^−^ within the biological matrix in negative-ion mode. This was only visible in the samples with ciprofloxacin added, so the peak presumably derives from ciprofloxacin or a ciprofloxacin metabolite (Fig. 5B).

The penetration of *P. aeruginosa* biofilms by tobramycin and ciprofloxacin has been studied by using labeled versions of the antibiotics (23). Labeled ciprofloxacin was found to readily penetrate while positively charged labeled tobramycin was sequestered at the biofilm periphery. However, labeling the antibiotics increased their MICs. The label-free imaging of antimicrobials at “real-life” concentrations with SIMS is a clear advantage over labeling experiments—the addition of a label to an antimicrobial compound may affect normal function. Fluorescent tagging is common with large macromolecules such as proteins but has obvious disadvantages when the fluorophore is as large as or larger than the target analyte, as is frequently the case with antibiotics. Some antimicrobials, such as gallium, cannot be fluorescently labeled at all. TOF-SIMS has previously been used to image endogenous antimicrobials in one species (3), to visualize the distribution of an antimicrobial compound within pig skin (30), and recently to analyze the localization of two antibiotics at high concentration at a single-cell level (31). However, the potential of SIMS for analyzing small molecules such as antibiotics in complex biological matrices has yet to be fully explored.

It was encouraging, therefore, that we were able to detect the parent ions of both ciprofloxacin and tobramycin by TOF-SIMS (Fig. 6) at real-life concentrations, even though they belong to very different chemical classes (fluoroquinolones and aminoglycosides, respectively). Clearly, analysis of antibiotics in biological matrices by SIMS will be more challenging—we could not detect tobramycin here and could detect ciprofloxacin only as the fluoride ion—but there is real potential for the future analysis of these compounds and other antibiotics as well.

### Endogenous bacterial compounds and an antimicrobial can be detected in the same sample.

**FIG 7:**
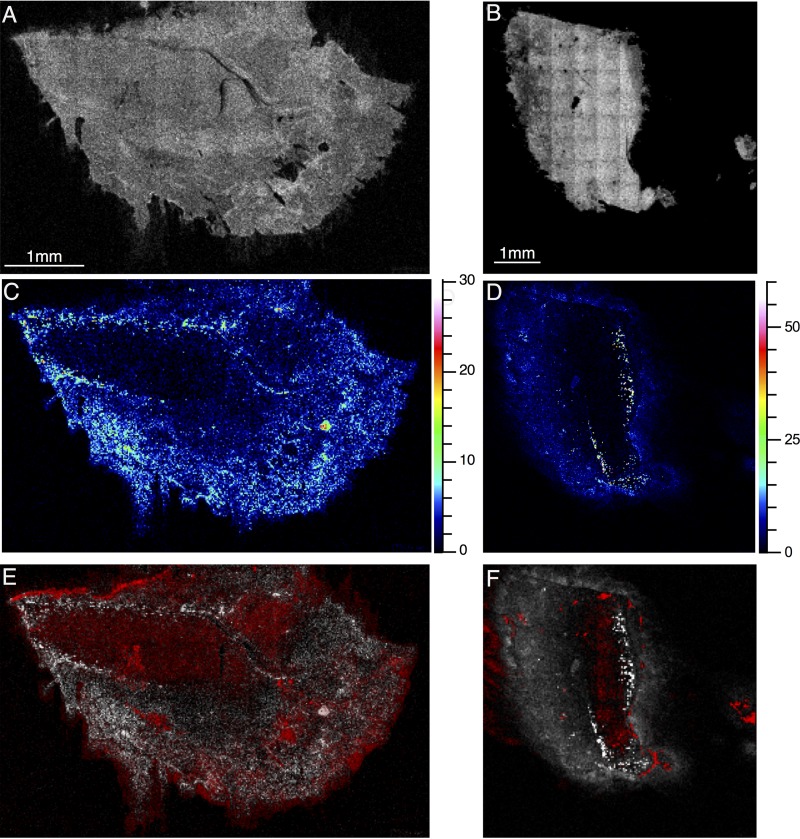
Ions representative of bronchial tissue (*m/z* 88.01; total cell count in panel A, 7.091 × 10^5^; total cell count in panel B, 1.434 × 10^6^) and NHQ (total cell count in panel C, 1.244 × 10^5^; total cell count in panel D, 1.819 × 10^5^) in sections of *P. aeruginosa*-infected bronchus from two distinct samples, both treated with 5 μg/ml gallium nitrate. Panels E and F show the distribution of an NHQ ion (white) and Ga^+^ (red) (total cell counts, 1.033 × 10^6^ and 2.083 × 10^6^ in panels E and F, respectively).

We wanted to look at the penetration of a biofilm by an antimicrobial. Gallium and ciprofloxacin peaks were clearly present in all scans throughout sputter depth profiling through to the ITO substrate, suggesting that both were able to fully penetrate the biofilms grown on ITO-coated glass after a 5-min exposure time. As a result, we have not shown these data in a figure, as there is little spatial localization of the compounds—they are essentially present throughout the ITO-coated glass biofilms. This is likely due to relatively thin biofilms (Fig. 1), although previous evidence has also suggested that ciprofloxacin may penetrate thick biofilms rapidly too (23). The lung tissue model clearly allows the growth of more complex and realistic bacterial assemblies, both as biofilms and as surface-attached bacteria, even deep within a tissue sample. We exposed samples grown on bronchial sections to gallium nitrate for 15 min and mapped the distribution of Ga^+^ and NHQ in treated samples (Fig. 7). The two ions appear to be present in different regions of the tissue; areas with high NHQ concentrations have low concentrations of gallium and, on the basis of the assumption that NHQ represents biofilm formation, gallium appears not to penetrate areas where biofilm is present. Gallium has been shown to be effective in preventing the formation of biofilms (25) because of its chemical similarity to iron—it is substituted for iron ions by bacteria and so inhibits iron-dependent processes. Gallium may increase the antimicrobial action of other antibiotics in biofilms, but free gallium does not appear to be particularly effective against mature biofilms (32).

Gallium did not seem to readily penetrate the biofilm in the pig bronchus model after a 15-min exposure time. The ability of an antibiotic agent to penetrate a biofilm may not tell us anything about its efficacy (33), but penetration of the biofilm seems to be important for effective antimicrobials so that all cells are exposed to the antimicrobial agent.

There are two approaches for obtaining 3D images using TOF-SIMS. The first is with sectioning; 2D images of consecutive sections can be combined to give a 3D image, as is common in other types of mass spectrometry imaging, such as MALDI or DESI (34). The second approach is sputter depth profiling, an extremely useful technique for samples that are too small for cryosectioning, particularly microbial samples, single cells, or samples that cannot be sectioned without detrimental structural damage. In the present study, for example, we were unable to section samples grown on glass slides. An alternative technique, focused ion beam milling, has also been used in conjunction with TOF-SIMS analysis when sectioning is not feasible, for very small samples (35, 36). Sectioning and depth profiling have previously been used in combination to image *Bacillus thuringiensis* spores using NanoSIMS (37) to validate the use of depth profiling for these samples. Spores of around 2 by 0.9 μm were imaged after sectioning, as well as being analyzed using depth profiling. A combined approach is likely to be ideal in many cases—sectioning allows access to the inside of a sample on a larger scale, while sputtering allows further profiling on a micrometer scale. Sputtering may also be useful to remove any contaminants (for example, embedding medium) deposited on a section’s surface during cryosectioning (29).

### Conclusions.

We have used TOF-SIMS to detect exogenous, unlabeled antimicrobials in biological samples. We were able to image the interior of samples—both surface-attached biofilms and complex structures grown on biological tissue. We detected both biological metabolites (bacterial quorum-sensing quinolone molecules) and exogenous xenobiotics, including small-molecule antibiotics and Ga ions. The parent ions of the antibiotics were not detected in the biological matrices, and further work will be important to characterize a wider range of compounds, their realistic detection limits, and the utility of TOF-SIMS in analyzing antibiotics at relevant concentrations. Future developments in instrumentation are likely to improve the feasibility of working with these analytes. For analyzing biofilms, cryosectioning and depth profiling were both valuable techniques for 3D imaging, having different and complementary roles. TOF-SIMS has the potential to be a powerful tool for future analysis of bacterial infection, both in lab biofilm models and in more complex tissue samples.

## MATERIALS AND METHODS

### Biofilm growth on ITO-coated glass.

*P. aeruginosa* biofilms were grown on ITO-coated glass (Visiontek Systems, United Kingdom) to provide an optimal substrate for TOF-SIMS analysis. To encourage the growth of thick biofilms, we used a *P. aeruginosa retS* mutant strain (PA14 *retS*) that overproduces exopolysaccharides, leading to a hyperbiofilm phenotype (38). Cultures were grown overnight in LB medium and then diluted to an optical density at 600 nm of 0.1 in LB. Pieces of ITO-coated glass approximately 1 cm^2^ in area were sterilized with ethanol and rinsed in sterile water before being placed individually in a sterile 24-well plate. Diluted culture (1 ml) was added to each well, and plates were incubated with shaking at 37°C to allow biofilm formation. After 96 h, biofilms were removed from liquid culture and placed in a new well and M63 medium containing both gallium(III) nitrate (5 μg/ml) and ciprofloxacin (20 μg/ml) or tobramycin (10 μg/ml) was added. Antibiotics were added at 20 times the MIC, calculated by a 2-fold dilution method. Samples were exposed to the antimicrobial mixture for 10 min and then quick-frozen by placing the glass pieces on an aluminum block precooled to −80°C; this was then transferred to a freeze-drier and lyophilized. Rapid freezing is important to preserve the structural integrity of biological samples; plunging samples into liquid nitrogen is well known to result in insufficiently fast freezing because of the Leidenfrost effect. Freezing in cooled liquids (e.g., liquid nitrogen-cooled isopentane) is a better alternative, but we worried that this might affect the delicate biofilms. Freeze-clamping is a standard technique for rapid freezing of tissues, using tongs with cooled aluminum plates; our current technique does not use freeze-clamping tongs but provides for rapid freezing by direct contact of the slide with a cooled metal block. Scanning electron microscopy (SEM) was used to confirm that the cells were growing in biofilms on the glass slides. Images were taken with a JSM 5610 LV microscope at 20 kV (×50 magnification) and a FEI Helios DualBeam NanoLab 600 system at 5 kV (×250 and ×10,000 magnifications).

### Pig lung model.

Pig lungs were purchased from a local butcher and prepared at the University of Nottingham. Lung sections were prepared essentially as described by Harrison and Diggle (39). To minimize contamination, the area to be dissected was briefly seared with a hot palette knife before dissection. Sections of bronchi (approximately 5 by 5 mm in area and around 2 mm in thickness) were dissected using a sterile razor blade, washed three times in cell culture medium, and then finally washed in artificial sputum medium (ASM) (40). Lung tissue pieces were placed in a sterile 24-well plate, each well of which contained 400 μl of ASM set with 0.8% agarose, and covered with 500 μl of liquid ASM. To minimize the growth of bacteria present in the lung tissue, 50 μg/ml ampicillin was added to the medium.

A sterile 30-gauge needle was used to inoculate each piece of tissue—after lightly touching a *P. aeruginosa* PA14 colony grown on LB agar, the needle was used to prick the lung tissue. A sterile needle was used to prick the lung tissue for mock infection controls. The inoculated tissue was incubated for 4 days at 37°C. Following incubation, samples were rinsed in phosphate-buffered saline (PBS) before being transferred to wells containing either PBS (controls) or PBS containing 5 μg/ml gallium nitrate for 15 min, and then snap-frozen in liquid nitrogen and stored at −80°C. Frozen samples were cryosectioned by the Pathology Core Service (Blizard Institute, Barts and the London School of Medicine and Dentistry, London), without embedding medium (because of potential interaction of commonly used embedding medium with SIMS analysis) to give 8-μm sections for TOF-SIMS analysis.

### TOF-SIMS.

Samples were analyzed with a TOF.SIMS 5 secondary-ion mass spectrometer (IONTOF GmbH, Münster, Germany). Biofilms grown on an ITO-coated glass substrate were analyzed with a 10-keV Ar1249 GCIB with a current of approximately 4 nA for controlled sputtering of the sample over a 500- by 500-μm area. Data were collected over a 200- by 200-μm area in HCBM (high-current bunched mode) with a 25-keV Bi_3_^+^ analysis beam over an *m/z* range of 0 to 800. The surfaces of sectioned pig bronchus samples were analyzed across the entire section of 6.5 by 5.5 mm with 250 pixels/mm using the Bi_3_^+^ primary ion gun in BAM (burst alignment mode). ITO-coated glass samples were analyzed in positive- and negative-ion modes, and bronchus samples were analyzed in positive-ion mode only. Standard spectra were obtained using solid standard compounds on double-sided tape attached to the sample platform. The calibration peaks used were CH_3_^+^, C_3_H_7_^+^, C_4_H_8_^+^, C_9_H_9_^+^, C_4_H_7_O^+^, and C_9_H_17_,N_2_^+^ in positive-ion mode and CH_2_^−^, OH^−^, NO_2_^−^, C_4_H^−^, and C_6_H_3_O^−^ in negative-ion mode. Flat substrate correction was applied to ITO-coated glass samples for 3D reconstruction using Sn^+^ as the representative substrate ion. For pig lung model images, 16-pixel binning was applied.

### Data availability.

The data generated in this study can be downloaded from the Open Science Framework repository at https://osf.io/329nx/.
